# The population genomics of adaptive loss of function

**DOI:** 10.1038/s41437-021-00403-2

**Published:** 2021-02-11

**Authors:** J. Grey Monroe, John K. McKay, Detlef Weigel, Pádraic J. Flood

**Affiliations:** 1 Department of Molecular Biology, Max Planck Institute for Developmental Biology, 72076 Tübingen, Germany; 2 Department of Plant Sciences, University of California Davis, Davis, CA 95616 USA; 3 College of Agriculture, Colorado State University, Fort Collins, CO 80523 USA; 4 Department of Plant Developmental Biology, Max Planck Institute for Plant Breeding Research, 50829 Cologne, Germany; 5 Department of Plant Breeding, Wageningen University, Wageningen, The Netherlands

**Keywords:** Rare variants, Molecular evolution, Evolutionary biology, Population genetics, Genome evolution

## Abstract

Discoveries of adaptive gene knockouts and widespread losses of complete genes have in recent years led to a major rethink of the early view that loss-of-function alleles are almost always deleterious. Today, surveys of population genomic diversity are revealing extensive loss-of-function and gene content variation, yet the adaptive significance of much of this variation remains unknown. Here we examine the evolutionary dynamics of adaptive loss of function through the lens of population genomics and consider the challenges and opportunities of studying adaptive loss-of-function alleles using population genetics models. We discuss how the theoretically expected existence of allelic heterogeneity, defined as multiple functionally analogous mutations at the same locus, has proven consistent with empirical evidence and why this impedes both the detection of selection and causal relationships with phenotypes. We then review technical progress towards new functionally explicit population genomic tools and genotype-phenotype methods to overcome these limitations. More broadly, we discuss how the challenges of studying adaptive loss of function highlight the value of classifying genomic variation in a way consistent with the functional concept of an allele from classical population genetics.

## The historical context

**Fig. 1:**
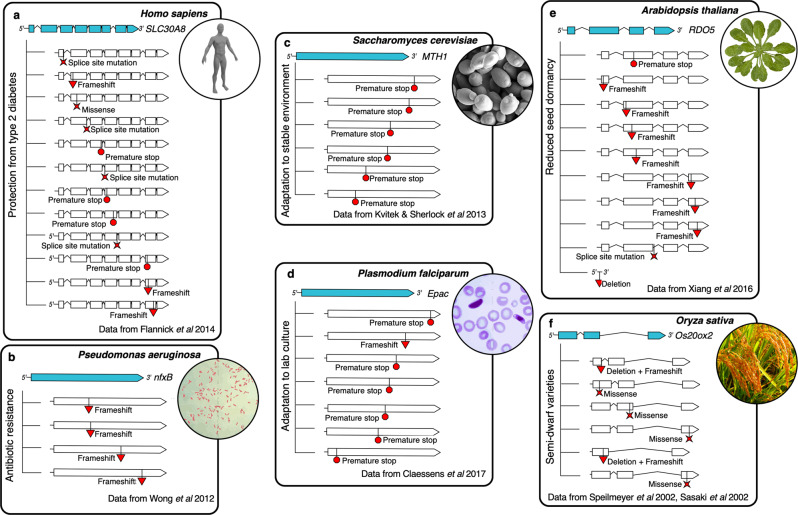
Examples of genes from different species with adaptive or beneficial loss-of-function alleles. In each example, multiple independent variants can be combined to constitute the population scale loss-of-function allele state. **a** Loss of function in *SLC30A8* is associated with reduced risk of type 2 diabetes in humans (Flannick et al. 2014; Dwivedi et al. 2019). **b** Experimental evolution in *Pseudomonas aeruginosa* resulted repeatedly in loss-of-function mutations in *nfxB*, conferring antibiotic resistance (Wong et al. 2012). **c** Experimental evolution in yeast led to consistent disruption of specific signaling pathway genes including *MTH1* during adaptation to stable environments (Kvitek and Sherlock 2013). **d** Populations of *Plasmodium falciparum* repeatedly evolved loss-of-function alleles in *Epac* during adaptation to lab culture environments (Claessens et al. 2017). **e** Natural *RDO5* loss-of-function variants in *Arabidopsis thaliana* occurred at high frequency in northwest Europe and caused reduced seed dormancy, a trait under strong locally adaptive selection (Xiang et al. 2016). **f** Adaptation to agricultural intensification led to selection for semi-dwarf rice, which is caused by loss-of-function variants in GA20ox2 (Spielmeyer et al. 2002; Sasaki et al. 2002).

Views on loss-of-function mutations—those abolishing a gene’s biomolecular activity—have changed considerably over the last half century. Early theories of molecular evolution that emerged during the 1960’s and 1970’s saw little potential for loss-of-function mutations to contribute to adaptation (Maynard Smith 1970). Except in the case of inactivated gene duplicates, nonfunctional alleles were often assumed to be lethal, with adaptation being generally regarded as a process explained only by the fixation of single, mutationally rare alleles that improved or altered a gene’s function (Orr 2005). Only relatively recently, through discoveries enabled by the availability of molecular sequence data, were alternative views of adaptive loss-of-function alleles formalized, most notably with the “less is more” ideas proposed by Olson (1999). Classical paradigms of molecular evolution had by that time been challenged, for example, by evidence that natural loss-of-function variants of CCR5 lead to reduced HIV susceptibility in humans (Libert et al. 1998). Discoveries during the subsequent two decades have continued to support the idea that loss of function contributes to adaptation (Murray 2020), with cases of adaptive or beneficial loss of function being discovered across diverse organisms, genes, traits, and environments (Fig. 1).

Today, reductive genome evolution is viewed as a powerful force of adaptation (Wolf and Koonin 2013) and gene loss is considered an important source of adaptive genetic variation (Albalat and Cañestro 2016; Murray 2020). The flood of -omics data generated in recent years is beginning to reveal the extent of loss of function and gene content variation segregating within species. Pan-genome and pan-transcriptome analyses have found that gene absence variation is pervasive in both prokaryotic and eukaryotic species (Jin et al. 2016; McInerney et al. 2017; Gerdol et al. 2020). And surveys of functional genomic diversity in organisms from *Arabidopsis thaliana* (Monroe et al. 2018; Xu et al. 2019) to humans (MacArthur et al. 2012; Karczewski et al. 2020) have revealed extensive genetic variation causing predicted loss of function. Yet, the adaptive importance of such variation remains largely unknown.

While the existence of adaptive loss of function is no longer seriously disputed, the assumed maladaptive nature of loss of function from early theories can persist in the language of population genetics such as in the continued use of *deleterious* as a synonym for *loss-of-function* (Moyers et al. 2018). Perhaps less visible but more consequential, historical assumptions about loss of function remain implicit in some analyses of DNA sequence variation as many classic tests for evidence of selection or causal relationships with phenotypes are built upon expectations of adaptation only involving hard sweeps of single mutationally rare alleles (Pennings and Hermisson 2006a, b). Contemporary disagreements in population genetics can also reflect differences in views on the functional molecular basis of adaptation. This can be seen for instance in alternative perspectives on the relative importance of soft versus hard selective sweeps, a debate which is inherently connected to the propensity for adaptation to involve recurrent loss-of-function mutations (Messer and Petrov 2013; Jensen 2014).

**Fig. 2:**
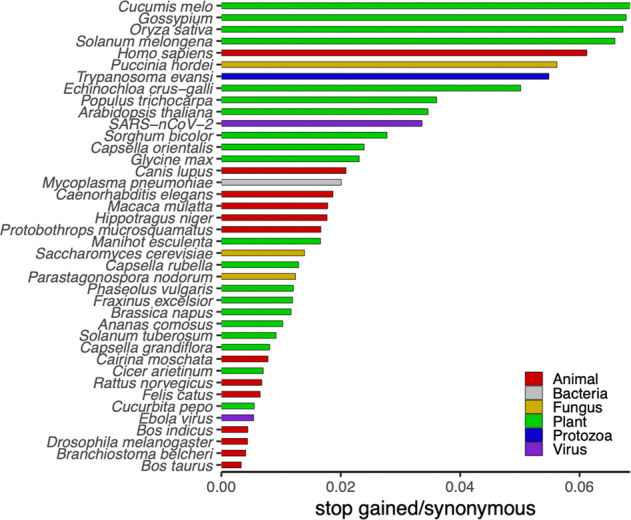
Rates of putative loss-of-function variants (stop gained) relative to synonymous single nucleotide polymorphisms reported in recent whole genome re-sequencing projects (Table 1). Species exhibit considerable differences in the ratio of stop gained to synonymous single nucleotide polymorphisms, with a 20-fold difference between the greatest (*Cucumis melo*) and fewest (*Bos taurus*) observations. The causes for these differences between species and, more generally, the (mal)adaptive nature of this extensive loss-of-function variation remain largely unknown.

**Fig. 3:**
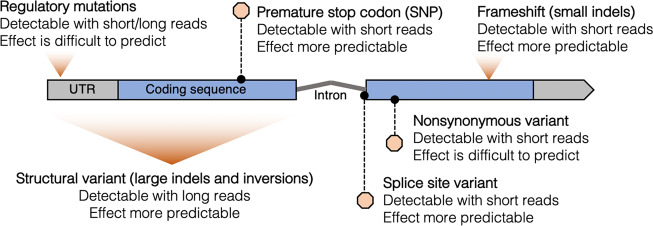
Examples of loss-of-function (LoF) variants. Shown are those types caused by different kinds of mutations (SNP single nucleotide polymorphism, indel insertions and deletions), which vary in the kind of data needed to detect them (short/long read sequencing) and the predictability of their effect on gene function.

**Fig. 4:**
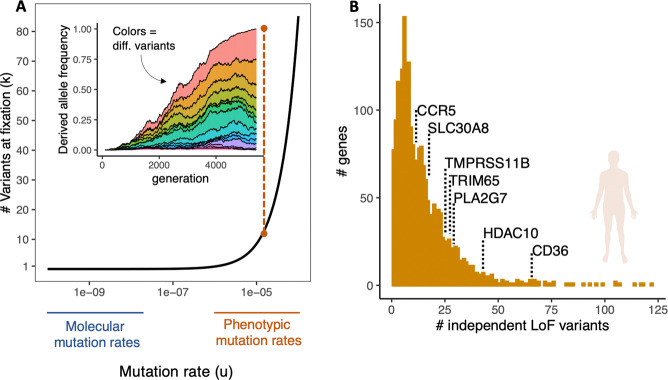
Theoretical predictions and empirical observations of allelic heterogeneity. **a** Predicted values of the number of independent variants of the same allele observed at fixation (*k*) as a function of mutation rates (*u*). Equation based on Haldane (1927) and Kimura (1962) and taken from Wilson et al. (2014). Predictions are based on an effective population size (*N*_*e*_) of 50,000 and selection coefficient (s) of 0.01. Highlighted are frequently observed ranges of empirical estimates of mutation rates from classic population genetics (Muller 1928; Haldane 1933; Rhoades 1941; Stadler 1946, 1948) and sequence-based mutation rates from modern molecular genomics (Lynch et al. 2016). Inset figure illustrates the hypothetical dynamics of multiple independent alleles (each a different color) with positive selection. Collectively the variants increase in frequency, ultimately leading to fixation of adaptive variants (elimination of deleterious ancestral allele), but individually each variant remains at low frequency. **b** Detected levels of allelic heterogeneity in genes enriched for loss of function in humans (obs > exp) reported by (Karczewski et al. 2020). Highlighted are cases of previously studied genes with evidence of beneficial effects or positive selection CCR5 (Libert et al. 1998), SLC30A8 (Flannick et al. 2014; Dwivedi et al. 2019), TMPRSS11B (Updegraff et al. 2018), TRIM65 (Wang et al. 2016; Wei et al. 2018), PLA2G7 (Song et al. 2012), HDAC10 (Dahiya et al. 2020), CD36 (Fry et al. 2009; Love-Gregory et al. 2011).

**Fig. 5:**
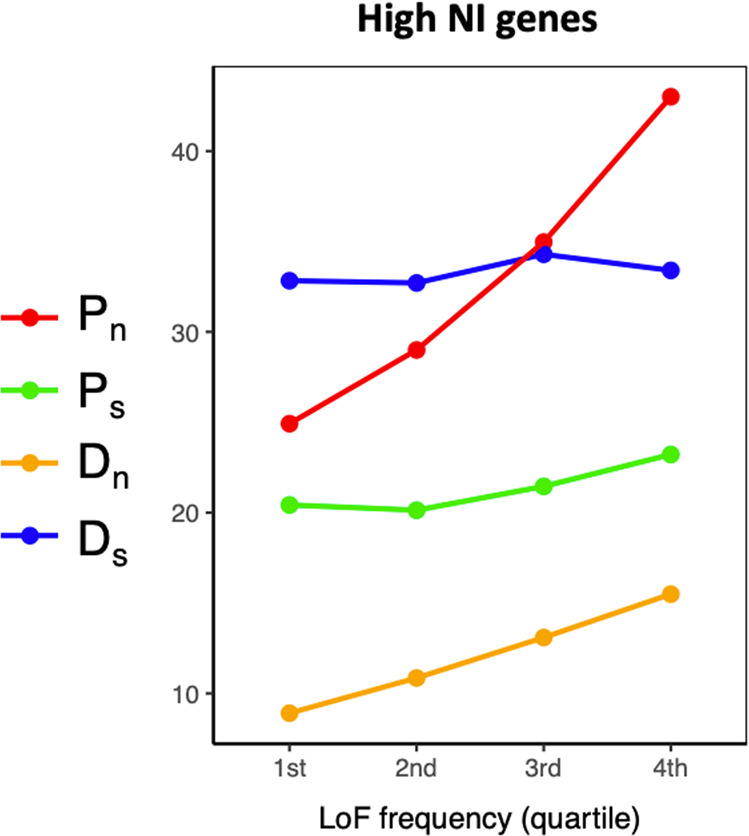
Among genes with high Neutrality Index (*NI*), those with a high frequency of loss-of-function alleles are enriched for non-synonymous polymorphism (*P*_*n*_). Shown here are mean components of *NI* in *A. thaliana* genes with high (top 20%) *NI* values in relation to loss-of-function (LoF) allele frequencies (binned by quartiles). Loss-of-function calls based on approach from (Monroe et al. 2018; Baggs et al. 2020) and data from (Monroe et al. 2020). *P*_*n*_ = non-synonymous polymorphism, *P*_*s*_ = synonymous polymorphism, *D*_*n*_ = non-synonymous divergence, *D*_*s*_ = synonymous divergence (using *A. lyrata* as an outgroup).

**Fig. 6:**
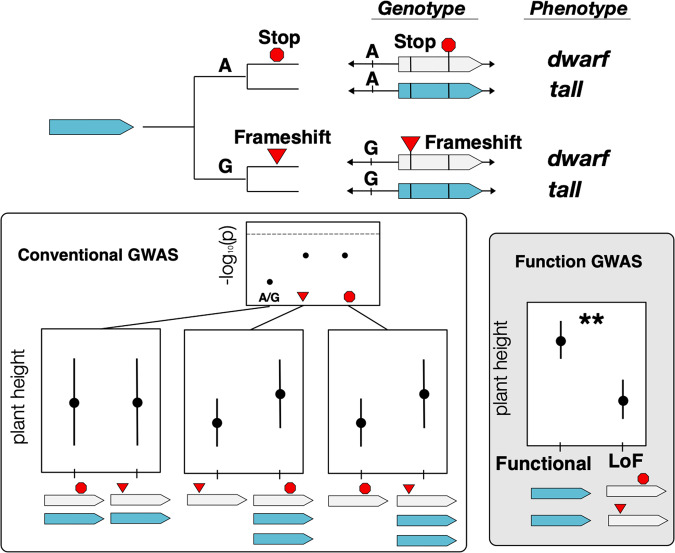
Imagined case in which independent loss-of-function (LoF) alleles give rise to adaptive dwarf phenotypes inspired by (Barboza et al. 2013). In this case, a premature stop codon and a frameshift mutation have arisen in alternative genetic backgrounds distinguished here by a nearby SNP (top). Conventional, functionally agnostic GWAS (bottom left) tests for association between individual variants and the trait of interest, in this case plant height, fail because none of the individual variants capture the functionally definitive variation (indicated by p-values below the significance threshold marked by the dashed line). An alternative approach, functional GWAS, first annotates variants according to predicted functional effects, then defines alleles as functional or non-functional. This corrects for allelic heterogeneity when testing for allele-trait associations and results in a significant allele trait association (bottom right).

The aim of this article is to examine theoretical and empirical advances describing the population evolutionary dynamics of beneficial loss-of-function alleles, which remain on one hand a low-hanging fruit when it comes to functionally classifying molecular diversity but on the other, a particularly challenging class of molecular variants to study using common population genetics models. We hope this review will facilitate new considerations of the population genomic diversity now being revealed with the widespread generation of whole genome sequence data (Table 1, Fig. 2). We also hope to shed light on some practical challenges confronting population geneticists (Figs. 3 and 4) and the intriguing dynamics of loss-of-function alleles through the lens of classic models (Fig. 5). We further discuss advances in sequencing technologies and annotation approaches that are facilitating new ways to discover cases of beneficial loss-of-function and more broadly, syntheses between modern genomics and the functional concept of alleles from classic population genetics (Fig. 6).

**Table 1:** Recent whole genome re-sequencing projects with functional annotations of variants. Numbers of premature stop, synonymous, and non-synonymous single nucleotide polymorphisms indicate number of variants segregating among the genotypes sequenced (Fig. 2). Sample Size = number of genotypes sequenced.

Species	Sample size	Premature stops	Synonymous	Non-synonymous	Citation
*Ananas comosus*	89	7,084	689,019	589,484	(Chen et al. 2019)
*Arabidopsis thaliana*	1,135	27,813	803,665	1,135,115	(1001 Genomes Consortium 2016)
*Bos indicus*	20	1,132	255,296	155,251	(Iqbal et al. 2019)
*Bos taurus*	15	3,837	1,155,244	524,103	(Zhang et al. 2019)
*Branchiostoma belcheri*	20	11,487	2,818,189	1,467,863	(Bi et al. 2020)
*Brassica napus*	991	1,413	120,926	79,018	(Wu et al. 2019)
*Caenorhabditis elegans*	330	5,084	271,950	261,538	(Cook et al. 2017)
*Cairina moschata*	15	285	36,517	19,817	(Gu et al. 2020)
*Canis lupus*	722	11,273	540,063	332,559	(Plassais et al. 2019)
*Capsella grandiflora*	15	5,209	644,326	478,238	(Koenig et al. 2019)
*Capsella orientalis*	16	269	11,250	13,281	(Koenig et al. 2019)
*Capsella rubella*	50	2,508	194,078	171,071	(Koenig et al. 2019)
*Cicer arietinum*	16	352	50,290	38,078	(Thudi et al. 2016)
*Cucumis melo*	1,175	7,030	102,687	94,426	(Zhao et al. 2019)
*Cucurbita pepo*	7	864	156,828	111,687	(Xanthopoulou et al. 2019)
*Drosophila melanogaster*	205	1,532	351,255	182,520	(Huang et al. 2014)
*Ebola virus*	140	3	555	403	(Ladner et al. 2015)
*Echinochloa crus-galli*	328	9,264	184,746	319,816	(Ye et al. 2019)
*Felis catus*	54	838	128,844	77,662	(Buckley et al. 2020)
*Fraxinus excelsior*	37	2,997	251,249	259,946	(Sollars et al. 2017)
*Glycine max*	1,007	2,826	122,469	182,479	(Torkamaneh et al. 2019)
*Gossypium* spp.	243	6,851	101,059	128,512	(Du et al. 2018)
*Hippotragus niger*	7	201	11,350	11,386	(Koepfli et al. 2019)
*Homo sapiens*	141,465	133,019	2,173,110	4,548,307	(Karczewski et al. 2020)
*Macaca mulatta*	133	2,642	148,278	126,445	(Xue et al. 2016)
*Manihot esculenta*	203	4,399	265,094	299,197	(Ramu et al. 2017)
*Mycoplasma pneumoniae*	15	88	4,382	6,891	(Xiao et al. 2015)
*Oryza sativa*	3,024	198,609	2,952,705	3,599,083	Wang et al. 2018)
*Parastagonospora nodorum*	197	2,815	226,803	160,159	(Richards et al. 2019)
*Phaseolus vulgaris*	683	1,352	112,173	97,536	(Wu et al. 2020)
*Populus trichocarpa*	1,014	8,365	231,894	333,036	(Piot et al. 2019)
*Protobothrops mucrosquamatus*	22	883	53,023	56,553	(Aird et al. 2017)
*Puccinia hordei*	5	2,629	46,763	67,526	(Chen et al. 2019)
*Rattus norvegicus*	40	285	42,182	26,239	(Hermsen et al. 2015)
*SARS-nCoV-2*	8,053	90	2,678	4,731	(Rayko and Komissarov 2020)
*Saccharomyces cerevisiae*	1,011	7,207	517,729	549,300	(Peter et al. 2018)
*Solanum melongena*	7	438	6,645	12,828	(Gramazio et al. 2019)
*Solanum tuberosum*	201	4,962	541,208	515,492	(Li et al. 2018)
*Sorghum bicolor*	44	3,114	112,255	112,108	(Mace et al. 2013)
*Trypanosoma evansi*	15	1,685	30,714	53,002	(Lazaro et al. 2020)

## What are loss-of-function alleles?

Classifying biological diversity into discrete categories has always been difficult. Descriptions of even the most fundamental biological units such as cell types, populations, and species can be challenging. Yet these classifications provide units of study that help make sense of biological and evolutionary phenomena. This is also true for alleles.

The existence of a category of alleles distinguished by a derived loss of biochemical function has been described by various names: “amorphic” (Muller 1932), “loss-of-function” (Jones 1972), “nonfunctional” (Nei and Roychoudhury 1973), “knockout” (Kulkarni et al. 1999),”null” (Engel et al. 1973), “pseudogene” (Jacq et al. 1977), or simply “gene loss” (Zimmer et al. 1980). Total gene loss is the most obvious case of loss of function. Comparisons of gene content between distantly related species have revealed considerable evidence for adaptation via complete deletion of genes or even entire sets of functionally related genes (Wang et al. 2006; Blomme et al. 2006; Will et al. 2010; McLean et al. 2011; Griesmann et al. 2018; van Velzen et al. 2018; Sharma et al. 2018; Huelsmann et al. 2019; McGowen et al. 2020; Baggs et al. 2020). Pangenome analyses have revealed extensive gene content variation segregating within species. For example, the average *Brachypodium distachyon* genotype is missing almost half of the genes observed in the species pangenome (Gordon et al. 2017). Yet total gene loss is not the only means by which loss of function can occur. In their review of evolution by gene loss, Albalat and Cañestro (2016) point out that single mutations and many mutation types such as premature stop codons, frameshifts, splice site disruptions, and elimination of regulatory regions required for gene expression can have effects that are functionally indistinguishable from complete gene loss. Here we will discuss how the phenomenon of allelic heterogeneity—that numerous types of mutations can produce the same functionally analogous allele—is important for understanding the evolutionary dynamics and implications of adaptation by loss of function.

First principles and empirical evidence indicate that many types of mutations can have effects that are equivalent to total gene loss, and for the purposes of this review, we employ this definition of complete gene losses being functionally equivalent to other loss-of-function mutations such as premature stop codons. However, there is the practical difficulty that these different types of mutations vary in how easily they can be detected and correctly annotated as loss-of-function alleles (Fig. 3). Insertions and deletions that interrupt the reading frame of a protein coding region (frameshift mutations), for example, might be readily classified as loss-of-function alleles because the downstream amino acid sequence will be severely disrupted. Yet a frameshift mutation at the extreme 3′ end of a coding region affecting only a few amino acids might be functionally distinct from a frameshift mutation at the extreme 5′ end disrupting the entire coding sequence. One simple heuristic to address this ambiguity is a threshold, measured by the portion of the gene affected by functionally disruptive mutations, at which an allele is classified as loss-of-function. This approach can be used to classify premature stop codons, frameshift, splice site disruptions, start loss, and inframe insertions and deletions. In humans (MacArthur et al. 2012; Karczewski et al. 2020) and *Arabidopsis thaliana* (Monroe et al. 2018; Baggs et al. 2020), loss-of-function mutations affecting only a small fraction (e.g., <10%) of total coding sequence in a gene were ignored when classifying loss-of-function variants. Such cutoffs are supported by the observation that even in genes not thought to be experiencing adaptive loss of function, there is an enrichment for otherwise predicted loss-of-function mutations that affect only small fractions of coding regions (MacArthur et al. 2012; Flowers et al. 2009), suggesting reduced functional impact of such variants.

Other single mutations causing loss of function may be even more difficult to predict. Mutations changing functionally critical amino acids can disable a protein’s molecular function. Indeed, detailed studies of individual genes have uncovered non-synonymous loss-of-function variants (Sasaki et al. 2002; Barboza et al. 2013; Zhang and Jiménez-Gómez 2020; Song et al. 2020, 2014) suggesting the maintenance of extensive cryptic (not easily identified as loss-of-function from standard annotation pipelines) genetic loss-of-function variation within populations (Table 1). But identifying non-synonymous mutations which result in loss-of-function among the numerous non-synonymous polymorphisms is difficult since experimental validation of the functional impacts for every non-synonymous variant is infeasible at genomic scales. Instead, predictions of functional impact must be predicted by more sophisticated methods (Tang and Thomas 2016), such as quantifying changes in the chemical properties of amino acid substitutions (Grantham 1974), sequence homology (Ng and Henikoff 2001), known phenotypic effects (Schwarz et al. 2010), the context of known domains and protein structures, or through integration of multiple methods with tools such as CADD (Kircher et al. 2014; Tang and Thomas 2016). Emerging statistical machine learning approaches, such as unsupervised latent variable models can also detect otherwise cryptic loss of function caused by non-synonymous substitutions (Riesselman et al. 2018). The effect of coding sequence variation on protein folding may also be predicted from deep learning approaches, such as AlphaFold (Senior et al. 2020). Beyond mutations affecting coding sequence, eliminating gene expression could also cause loss of function (Albalat and Cañestro 2016), but identifying such mutations is challenging and validation at genomic scales is currently difficult. However, as with non-synonymous substitutions, advances in machine learning have also led to models that can predict functional consequences of non-coding variants (Zhou and Troyanskaya 2015). These methods can also be used to predict variants causing loss of gene expression. The application of these new tools presents a path forward for a new generation of functionally explicit analyses of genomic diversity. More broadly, a major goal of modern biology is to predict molecular function from genomic sequence data. The study of adaptive loss-of-function alleles could serve as a model class of genetic variation to spearhead this effort.

The accurate prediction of allele function from population genomic data assumes that researchers have complete information about what is functional and about sample sequence diversity. The genomes of reference genotypes used as the basis of comparison for whole genome re-sequencing projects can themselves already harbor loss-of-function alleles, obfuscating definitions of “functional” and therefore loss of function as well. For example, the standard *A. thaliana* reference is based on the genome of the Col-0 genotype, which is known to harbor an adaptive loss-of-function variant in the vernalization gene *FRIGIDA* (Johanson et al. 2000). Therefore, to study natural functional variation in this locus, Zhang and Jiménez-Gómez (2020) computationally swapped the reference sequence at this locus with a known functional allele and remapped public short read sequencing data to discover novel loss-of-function variants. Such scenarios at genome-wide scales motivate ongoing efforts to generate and annotate multiple reference genomes for a given species (Michael et al. 2018; Sun et al. 2018; Yang et al. 2019; Jiao and Schneeberger 2020; Zhou et al. 2020; Liu et al. 2020; Michael and VanBuren 2020; Li et al. 2020) to be used as a basis of comparison to describe broader population genetic diversity. Furthermore, most population-scale genome sequencing has been completed using short read sequencing technologies (</= 300 base pairs), which require greater depth to reliably detect small insertions and deletions (compared to single nucleotide polymorphisms) and may be unable to reliably detect large insertions, deletions, and other structural variants altogether (Kishikawa et al. 2019). These unseen variants could be a considerable source of loss-of-function alleles in natural populations, and the difficulty to detect them (Fig. 3) might imply that many adaptive loss-of-function alleles are yet to be discovered. Thus, most assessments of population genetic variation are still limited to only a fraction of functional allelic diversity. Third-generation sequencing technologies are therefore facilitating more complete characterizations of allelic diversity (Alonge et al. 2020; Liu et al. 2020). Precise characterization of alleles at functional molecular resolutions is greater than being a technical challenge for studying sequence variation—it is essential for making sense of genomic sequence data through the lens of classic population genetics theory. We will see how this is exemplified in cases of adaptive loss-of-function alleles, whose high effective mutation rate leads to a breakdown of the assumptions underlying standard approaches used to detect signatures of selection and genotype to phenotype mapping.

## Many ways to break a gene: quantifying allelic heterogeneity

A characteristic feature of genes experiencing adaptive loss of function is the existence of multiple functionally equivalent variants. To understand the extent of such variation, we can quantify and predict allelic heterogeneity, the phenomenon where multiple independent molecular variants exist that produce functionally analogous alleles of a given locus (Haldane 1927; Kimura 1962; Wilson Petrov et al. 2014; Ralph and Coop 2015). Assuming a constant effective population size (***N***_***e***_), mutation rate of an adaptive allele (***u***), and selection coefficient on that adaptive allele (***s***), the expected number of mutationally independent alleles of the locus that will be observed in a population at the moment of allele fixation (***k***), a unit of allelic heterogeneity, is predicted (Wilson Petrov et al. 2014) as:

1 $$k = 2{\it{log}}\left( {N_eS} \right)N_eu$$

The expected number of independent alleles at fixation is directly correlated with the mutation rate (Eq. 1, Fig. 4). Early studies of mutation rate quantified the frequency at which mutations gave rise to a particular allelic state, defined by its phenotypic effect. These studies often reported phenotypic mutation rate estimates ranging from 10^-4^ to 10^-6^ mutations (change in phenotype) per generation (Muller 1928; Haldane 1933; Rhoades 1941; Stadler 1946, 1948). Estimates of molecular mutation rates at the DNA sequence level are generally orders of magnitude lower: 10^−8^ to 10^−10^ mutations (change in sequence) per site per generation (Lynch et al. 2016). A partial explanation for the discrepancy between the range of phenotypic and molecular mutation rate is the obvious fact that many different molecular mutations can give rise to the same phenotypically/functionally effective allele type. Loss-of-function mutations exemplify this reality. Because there are hundreds or thousands of different molecular mutations that can produce a suite of analogous loss-of-function alleles (e.g., any premature stop codon or differently sized deletions along much of the coding region of a gene), the aggregated mutation rate for loss of function is expected to be orders of magnitude greater than the molecular mutation rate. At such high effective mutation rates we should predict, given biologically reasonable population sizes and selection coefficients, the existence of considerable allelic heterogeneity (Fig. 4a), which appears consistent with empirical observations (Figs. 1 and 4b).

## Mixed signals in signatures of selection

Early genetics employed a functionally definitive concept of an allele. Alleles were treated as local units of inheritance based on their functional effect, observed at the phenotypic level (e.g., Rhoades 1938). As such, at locus a experiencing adaptive loss of function, the (potentially multiple) variants causing the adaptive trait should act collectively as a single allele, even if due to independent mutational events (Pennings and Hermisson 2006a, b). If, for example, this functionally identical set of variants experiences positive selection, it behaves like a single allele according to predictions of classic population genetic theory (Orr 2005)—increasing in frequency to fixation (Fig. 4a, inset). Indeed, foundational models of population genetics (Haldane 1927) accommodate recurrent mutation and predict that adaptation will often involve multiple independent mutational origins given realistic population sizes, selection coefficients, and mutation rates (Eq. 1, Fig. 4a). But if we encounter such cases through analyses of DNA sequence alone, we may be troubled to find that the sequence variants only exhibit the expected evolutionary dynamics of classical alleles when considered as aggregated functional units, but not when analyzed individually (Remington 2015).

Scenarios like these have been extensively studied in a broad manner, in order to detect signatures of soft sweeps of multiple independent variants. A number of approaches have been developed to study soft sweeps. These generally do not attempt to classify variants into functional allele categories but instead look for evidence of increased frequency of multiple rather than single haplotypes in a functionally agnostic fashion (Schrider and Kern 2016; Hermisson and Pennings 2017; Harris et al. 2018; Mughal and DeGiorgio 2019; Stern et al. 2019; Hartfield and Bataillon 2020; Garud et al. 2020). Nevertheless, it is interesting to note that extensive research into soft sweeps came only after increasing evidence of the potential adaptive value of loss-of-function alleles had been published (Pennings and Hermisson 2006a, b 2006). In contrast, hard sweeps of a single adaptive variant were described during the era predominated by the view that loss-of-function mutations were necessarily deleterious, and adaptation could only proceed through mutationally rare gain-of-function alleles (Maynard Smith and Haigh 1974). Such historical dynamics speak to the interconnectedness, intentional or otherwise, between ideas about the functional molecular basis of adaptation and advances in the development of population genetic models and theories.

Unfortunately, population genetic statistics based on the expectation that adaptive alleles are mutationally rare perform poorly when this assumption is violated. For example, statistics based on the site frequency spectrum such as Tajima’s D do not deviate from neutral expectations in a predictable fashion for adaptive alleles with multiple mutational origins (Pennings and Hermisson 2006a). Similarly, statistics based on linkage disequilibrium around adaptive loci, though they tend to perform better for soft sweeps, also appear neutral if the number of mutational origins of an adaptive allele is high enough (Hermisson and Pennings 2017). For adaptive loss of function, this may often be the case. More generalized methods of detecting soft selective sweeps from independent mutational origins, such as the H12 statistic developed by Garud and colleagues (Garud et al. 2015) might be useful for detecting adaptive loss of function. The reciprocal is also true—known cases of adaptive loss of function could serve as valuable models for testing the limits of test statistics intended to detect soft sweeps.

More functionally explicit statistics of allelic variation are now possible because of the availability of whole genomic sequence data. However, the application of functional test statistics to genes experiencing putatively adaptive loss-of-function can yield surprising results. For example, the Neutrality Index (*NI*) (McDonald and Kreitman 1991; Rand and Kann 1996) estimates histories of selection by comparing rates of within-species polymorphism and between-species divergence. It is more functionally explicit than many population genetics statistics—comparing putatively functionally impactful (non-synonymous) versus silent (synonymous) variation. Where *P*_*n*_ = non-synonymous polymorphism, *P*_*s*_ = synonymous polymorphism, *D*_*n*_ = non-synonymous divergence, *D*_*s*_ = synonymous divergence

2 $$NI = \left( {P_n/P_s} \right)/\left( {D_n/D_s} \right)$$

Traditional interpretations of the results are based on the assumption that adaptive variants will become fixed and therefore be observed as diverged (*D*_*n*_) from related species rather than polymorphic (*P*_*n*_) within the study species. When genes putatively experiencing adaptive loss of function are investigated, they are often found to have high *NI* values (Le Corre et al. 2002; Flowers et al. 2009; Will et al. 2010; Rose et al. 2012; Monroe et al. 2016), a pattern that seems paradoxical given that high *NI* values are commonly interpreted as evidence of purifying selection (Weinreich and Rand 2000). But when considered with the knowledge that non-synonymous variants can themselves cause loss of function, and given the likely independent mutational origins of loss of function, this result is consistent with expectations of an enrichment of non-synonymous polymorphism in genes with both high frequency of loss of function and high *NI* (Fig. 5).

Increasingly functionally precise statistics such as the sum frequencies of losses of function in a given gene across all variants (Albalat and Cañestro 2016) might better describe loss-of-function alleles than functionally agnostic test statistics or descriptions of individual variants. Accelerations in whole genome sequencing technologies and improved capacity to classify previously cryptic loss-of-function variants may facilitate a new generation of functionally definitive population genetic models and methods. This would not only be valuable for improving the capacity to understand the forces shaping intraspecific loss-of-function, but more generally promote a re-synthesis between studies of molecular sequence variation and the function-based conception of alleles from early population genetic theory.

## Functionally explicit genotype-to-phenotype mapping

To identify genes contributing to adaptive phenotypic variation, Genome Wide Association (GWA) scans in natural populations have become a popular alternative to conventional mapping in an experimental population derived from a bi-parental cross. GWA is normally implemented by testing for associations between individual DNA sequence variants in a population and the phenotype (or environmental gradient) of interest. This statistical framework can fail to detect causal loci in the presence of allelic heterogeneity because none of the individual variants are linked to a single causal variant—an assumption of single-locus two-allele population genetic models (Korte and Farlow 2013). This problem is exemplified by loss of function variation in which, with a few notable exceptions (Song et al. 2020), allelic heterogeneity is expected to be the norm (Pennings and Hermisson 2006a, b).

The case of the GA-20 oxidase gene in plants provides a useful illustration of these challenges. This well-studied gene is involved in gibberellin biosynthesis and loss of function produces semi-dwarf phenotypes in wild plants and crop varieties of the Green Revolution (Fig. 1f) (Spielmeyer et al. 2002; Sasaki et al. 2002; Jia et al. 2009; Barboza et al. 2013). While functional experiments have demonstrated that loss of this gene causes considerable reduction in plant height, and investigations of natural molecular variation in *A. thaliana* identified cases of likely loss-of-function differences between genotypes, a conventional GWA looking for loci explaining variation in plant height failed to detect the GA-20 oxidase locus in *A. thaliana* (Barboza et al. 2013). However, when all of the genotypes with predicted loss-of-function variants were collapsed into a single allele state and their heights contrasted with the genotypes containing predicted functional variants, the known highly significant effect on plant height was detected (Barboza et al. 2013). Without previous knowledge that this gene plays an important role in plant height, it would have been missed by conventional GWA. This experiment provides a cautionary tale as to how conventional GWA approaches can fail in the presence of allelic heterogeneity at causal loci. It also highlights the power of functionally explicit GWA approaches based on population genetic models that allow for allelic heterogeneity—using predictions about functional effects of individual variants to collapse variants into allele classes (in this case, loss-of-function vs functional) so that a functionally explicit contrast can be made (Fig. 6).

To date, such a framework has been primarily used in the study of rare variants (Wu et al. 2011; Pan and Shen 2011; Zhang et al. 2017) to identify rare deleterious loss-of-function alleles associated with disease phenotypes in humans (Zuk et al. 2014) but it could also be used to find beneficial and adaptive loss of function as well. For example, loss of function in *SLC30A8* was found to be strongly associated with decreased risk of type 2 diabetes when all loss-of-function variants were collapsed into a single allele state (Flannick et al. 2014) (Fig. 1a), thus identifying its protein product as a promising therapeutic target to treat diabetes (Dwivedi et al. 2019). With population whole-genome-sequence data becoming available in model and non-model species, this approach can now be readily applied by evolutionary biologists at genome wide scales to discover loss-of-function alleles contributing to phenotypic evolution in populations (Monroe et al. 2020).

A functionally explicit GWA framework may have value beyond scanning genomes for causal loss-of-function alleles. More broadly, it reflects a step toward representing genetic diversity as a matrix of functionally relevant genetic alleles rather than a matrix of DNA sequence variants. While loss of function is currently the easiest allele state to classify, we anticipate that more nuanced and precise allele categories can be identified through analyses of population genomic diversity with advances in sequence annotation. Ideally, these categories would specify the activity of an allele along a scale that reflects Muller’s original categories of amorphic, hypomorphic, hypermorphic, antimorphic, and neomorphic states (Muller 1932). In addition to facilitating discovery of causal loci, functionally explicit methods of population genomics could be useful for predicting quantitative traits (i.e., genomic prediction) and address the problem of missing heritabilities (Manolio et al. 2009) that has frustrated modern geneticists for over a decade (Eichler et al. 2010).

## Outlook and concluding remarks

Loss-of-function alleles were once often held up as a paragon of deleterious genetic variation. Today a more nuanced appreciation for their potential role in adaptation has emerged. This new paradigm inspires investigations into deeper questions about the causes and consequences of adaptation by genetic loss of function. For example: Do species or populations differ in their capacity to adapt via loss of function, and if so, why? Does the high effective mutation rate of loss-of-function alleles lead to bias in the probabilities of different evolutionary outcomes? What is the contribution of adaptive loss of function to the phenomena of antagonistic pleiotropy and reproductive isolation? How does adaptation by loss of function affect long term evolutionary trajectories of populations and future evolvability? Ongoing technical breakthroughs promise to scale up the study of loss-of-function alleles experiencing positive selection for population genomic research to address these questions. More broadly, these lines of research provide paths toward advancing tools and concepts that facilitate a continued synthesis between functional molecular genomics and classic population genetic theory.
